# A nomogram predicting the recurrence of hepatocellular carcinoma in patients after laparoscopic hepatectomy

**DOI:** 10.1186/s40880-019-0404-6

**Published:** 2019-10-11

**Authors:** Yang-Xun Pan, Jian-Cong Chen, Ai-Ping Fang, Xiao-Hui Wang, Jin-Bin Chen, Jun-Cheng Wang, Wei He, Yi-Zhen Fu, Li Xu, Min-Shan Chen, Yao-Jun Zhang, Qi-Jiong Li, Zhong-Guo Zhou

**Affiliations:** 1 State Key Laboratory of Oncology in South China; Collaborative Innovation Center for Cancer Medicine, Sun Yat-sen University Cancer Center, Guangzhou, 510060 Guangdong P. R. China; 2 Department of Liver Surgery, Sun Yat-sen University Cancer Center, 651 Dongfeng Road East, Guangzhou, 510060 Guangdong P. R. China; 3 Department of Public Health, Sun Yat-sen University, Guangzhou, 510080 Guangdong P. R. China; 4 Department of Molecular Medicine California Campus, The Scripps Research Institute, 10550, North Torrey Pines Road, La Jolla, CA 92037 USA

**Keywords:** Hepatocellular carcinoma, Laparoscopic hepatectomy, Recurrence, Nomogram, American Joint Committee on Cancer TNM classification, Barcelona Clinic Liver Cancer staging system, Hepatitis B surface antigen, Tumor thrombus, Tumor invasion

## Abstract

**Background:**

Patients with hepatocellular carcinoma (HCC) undergoing surgical resection still have a high 5-year recurrence rate (~ 60%). With the development of laparoscopic hepatectomy (LH), few studies have compared the efficacy between LH and traditional surgical approach on HCC. The objective of this study was to establish a nomogram to evaluate the risk of recurrence in HCC patients who underwent LH.

**Methods:**

The clinical data of 432 patients, pathologically diagnosed with HCC, underwent LH as initial treatment and had surgical margin > 1 cm were collected. The significance of their clinicopathological features to recurrence-free survival (RFS) was assessed, based on which a nomogram was constructed using a training cohort (*n* = 324) and was internally validated using a temporal validation cohort (*n* = 108).

**Results:**

Hepatitis B surface antigen (hazard ratio [HR], 1.838; *P *= 0.044), tumor number (HR, 1.774; *P *= 0.003), tumor thrombus (HR, 2.356; *P *= 0.003), cancer cell differentiation (HR, 0.745; *P *= 0.080), and microvascular tumor invasion (HR, 1.673; *P * =0.007) were found to be independent risk factors for RFS in the training cohort, and were used for constructing the nomogram. The C-index for RFS prediction in the training cohort using the nomogram was 0.786, which was higher than that of the 8th edition of the American Joint Committee on Cancer TNM classification (C-index, 0.698) and the Barcelona Clinic Liver Cancer staging system (C-index, 0.632). A high consistency between the nomogram prediction and actual observation was also demonstrated by a calibration curve. An improved predictive benefit in RFS and higher threshold probability of the nomogram were determined by receiver operating characteristic curve analysis, which was also confirmed in the validation cohort compared to other systems.

**Conclusions:**

We constructed and validated a nomogram able to quantify the risk of recurrence after initial LH for HCC patients, which can be clinically implemented in assisting the planification of individual postoperative surveillance protocols.

## Background

Hepatocellular carcinoma (HCC) is a leading cause of cancer deaths globally, ranked as the fifth common malignancy and the second leading cause of cancer-related mortality [1, 2]. According to several guidelines, hepatectomy is recommended as a curative treatment for patients with solitary liver cancer and well-preserved liver function [3, 4]. Despite curative resection, the long-term prognosis of HCC patients is still unsatisfactory, with an extremely high recurrence rate exceeding 70% at 5 years, even in patients with HCC of size ≤ 5 cm [5, 6]. It is believed that the limitations of hepatectomy result in potential HCC recurrence due to residual cancer in the remnant liver or the possibility of de novo HCC recurrence induced by hepatitis B virus infection [7]. To ensure the appropriate management of patients after HCC resection for optimal survival prolongation, data on clinical, surgical, and pathological characteristics should be conjointly used for accurate survival prognostication to optimize individualized treatment planning.

Recent studies on laparoscopic surgery have consistently shown comparable outcomes to conventional surgery for hepatectomy in treating HCC, meanwhile, laparoscopic hepatectomy (LH) also possesses the advantages of having minimal surgical invasiveness and faster recovery [8, 9]. Although the recurrence rates of patients who underwent laparoscopic and conventional hepatectomy are similar, with 3-year disease-free survival rates ranging from 72.5% to 50%, prognostic factors for recurrence in these two groups of patients differ because of differentiation in patient selection for surgery and the operating techniques used [8]. To this end, Li et al. [9], Nakagawa et al. [10], and Umeda et al. [11] developed scoring systems for patients subjected to conventional hepatectomy to evaluate their recurrence probabilities, based on clinical and pathological variables. To develop the most cost-effective, postoperative surveillance protocol, there is a need to stratify the risks of recurrence in HCC patients after LH. However, few scoring systems have focused on laparoscopic hepatectomy (LH) for predicting recurrence-free survival (RFS).

Currently, the 8th edition of the American Joint Committee on Cancer (AJCC) tumor-node-metastasis (TNM) staging system and the Barcelona Clinic Liver Cancer (BCLC) Classification are based on the pathological information, and the treatment regimens for HCC patients are established according to these staging systems [10, 11]. Several studies pointed out that patients who were allocated to the same treatment according to similar disease characteristics had completely different clinical outcomes [10, 12]. This indicated that the present staging systems are inadequate for predicting recurrence and do not accurately reflect the biological heterogeneity of HCC patients. Therefore, a comprehensive, easy-to-use tool able to estimate individual risk by incorporating pathological and clinical factors could serve as a valuable decision-making tool for clinicians.

The aim of this study was to formulate and validate a predictive model capable of predicting the RFS of HCC patients after LH which in turn can be used to guide individualized post-LH surveillance protocols.

## Methods

### Patients and clinicopathologic data

Clinical records of patients with primary HCC diagnosed pathologically between January 2013 and January 2018 were retrieved from the information system of our cancer center. All patients had received LH. The clinical characteristics, liver function tests, intraoperative and pathological outcomes were recorded during hospitalization. The inclusion criteria were as follows: (1) patients who underwent LH with surgical margin > 1 cm as initial treatment and did not receive any preoperative treatment [13]; (2) patients with clear pathological diagnosis of HCC after LH; (3) patients with complete clinicopathological and follow-up data; and (4) patients who recovered from the operation and survived for > 1 month postoperatively. In addition, patients were excluded if they met the following exclusion criteria: (1) the surgical margin < 1 cm; (2) non-HCC diagnosis according to postoperative pathology; (3) had LH conversion to open hepatectomy during the LH operation; (4) perioperative death; and (5) had missing clinical data. The tumor stage was evaluated according to the 8th edition of the AJCC TNM classification and the BCLC staging system [10, 14]. The study protocol was approved by the Clinical Research Ethics Committee of Sun Yat-sen University Cancer Center (SYSUCC, Guangzhou, China), and all patients provided written informed consent (B2019-129-01).

Clinicopathological data, including age, gender, hepatitis B surface antigen (HBsAg), total bilirubin, alanine aminotransferase (ALT), aspartate transaminase (AST), albumin (ALB), alpha-fetoprotein (AFP), carbohydrate antigen 19-9 (CA19-9), hemoglobin (HGB), platelet count, international normalized ratio for prothrombin time, hepatitis B virus-DNA (HBV-DNA) copy number, liver macronodular cirrhosis (irregular nodules with a variation greater than 3 mm in diameter), intraoperative blood loss, portal vein embolization, surgical procedure, tumor size, tumor multiplicity, tumor encapsulation, tumor boundary, tumor thrombus, cancer cell differentiation, 8th AJCC TNM stage, microvascular tumor invasion (MVI), BCLC stage, Child–Pugh score, hospital stay, and operative time, were collected.

### Follow-up and study endpoints

All HCC patients were advised to receive regular follow-ups after completion of the primary therapy according to clinical guidelines [3]. Patients were generally followed up every 3 months in the first 2 years and every 6 months thereafter if no evidence of recurrence appeared in the following 3 to 5 years. For each follow-up, serological and imaging examinations were performed, including serum AFP, liver function test, routine blood test, computed tomography (CT), to monitor lung metastasis, and magnetic resonance imaging (MRI), to monitor intrahepatic recurrence. RFS was defined as the time interval between the date of operation and the date of the diagnosis of recurrence. For patients without any evidence of recurrence, the last follow-up date was December 31, 2018.

### Statistical analysis

The prognostic factors for RFS were identified using the R software (version 3.5.2; https://www.r-project.org/). The difference between the training cohort and the validation cohort was compared. Chi-square test or Fisher’s exact test was used to compare categorical variables. Continuous variables with normal distribution were compared using the Student’s *t* test, or the Mann–Whitney *U* test was used for variables with abnormal distribution. RFS curves were depicted using the Kaplan–Meier method and compared using the log-rank test. Variables were converted to categorized variables for univariable analysis, and the factors that showed significant associations with recurrence in the univariate logistic models were subsequently included in the multivariate Cox regression model to identify independent prognostic factors through backward selection. All reported *P* values are two-sided, and *P *< 0.05 was considered significant, unless stated.

### Nomogram

The patients were divided into the training and validation cohorts. After significant factors related to RFS in the training cohort were identified through multivariate analyses (*P* < 0.10), a nomogram for predicting the 1-, 2-, and 3-year RFS was constructed using the package of rms in R version 3.5.2 (http://www.r-project.org/). To quantify the discrimination performance of the proposed nomogram, the Harrell’s C-index was evaluated. In brief, a C-index value greater than 0.750 is considered to represent relatively good discrimination of the model. Calibration was performed by comparing the RFS probability with the Kaplan–Meier estimator. In the validation cohort, according to the established nomogram, the C-index and calibration curve were derived based on regression analyses. The receiver operating characteristic (ROC) curve was used to compare the proposed prediction model with the 8th AJCC TNM classification [14] and the BCLC staging system [11].

## Results

### Clinicopathologic characteristics of training and validation cohorts

In total, 432 patients who underwent LH for primary HCC were included in this study, and 9 patients were excluded because of intraoperative laparoscopic conversion to open hepatectomy. The median follow-up time for the entire cohort was 14.38 (4.60–29.38) months. For the nomogram construction and validation, we assigned patients treated between September 2014 and January 2018 (*n *= 324) to the training cohort and patients treated between January 2013 and August 2014 (*n *= 108) to the validation cohort. The clinical demographics of the training and validation cohorts during the perioperative period are summarized in Table 1. As for the overall cohort, 87.73% of the patients were male, the mean age was 52.00 (43.00–60.00) years, 99.07% patients were classified as Child–Pugh class A (scores 5 and 6), and 85.42% patients were HBsAg-positive, 74.31% patients were diagnosed as having liver macronodular cirrhosis with “light” as the predominant cirrhosis level (*n *= 304, 70.37%). The mean AFP and CA19-9 levels were 91.45 (6.39–1705.00) ng/mL and 19.24 (10.44–33.99) ng/mL, respectively. The mean operation time was 157.32 ± 58.95 min, and 143 (33.10%) patients received hepatic portal vein embolization to reduce bleeding. There were no significant differences in clinicopathological characteristics between the training and validation cohorts (Table 1).

**Table 1:** Baseline clinicopathologic characteristics for the training and validation cohorts of patients who underwent laparoscopic hepatectomy for hepatocellular carcinoma

Variable	Overall cohort (*n* = 432)	Training cohort (*n* = 324)	Validation cohort (*n* = 108)	*P* value^†^
Age [years; median (IQR)]	52.00 (43.00–60.00)	51.00 (43.00–60.00)	52.50 (44.00–60.25)	0.683
Sex [cases (%)]				0.933
Male	379 (87.73)	285 (87.96)	94 (87.04)	
Female	53 (12.27)	39 (12.04)	14 (12.96)	
Hepatitis B surface antigen [cases (%)]				0.937
Present	369 (85.42)	276 (85.19)	93 (86.11)	
Absent	63 (14.58)	48 (14.81)	15 (13.89)	
Child–Pugh score [cases (%)]				0.931
5	359 (83.10)	268 (82.72)	91 (84.26)	
6	69 (15.97)	53 (16.36)	16 (14.81)	
7	4 (0.93)	3 (0.93)	1 (0.93)	
AFP [ng/mL; median (IQR)]	91.45 (6.39–1705.00)	93.89 (6.79–1573.50)	78.82 (5.00–2231.75)	0.482
CA19-9 [ng/mL; median (IQR)]	19.24 (10.44–33.99)	20.18 (11.05–34.42)	15.96 (8.58–30.32)	0.692
AST (IU/L; mean ± SD)	41.50 ± 33.02	41.88 ± 35.45	40.36 ± 24.43	0.680
ALT (IU/L; mean ± SD)	41.93 ± 34.14	43.25 ± 37.22	38.00 ± 22.18	0.167
Hemoglobin (g/L; mean ± SD)	154.56 ± 193.60	157.11 ± 223.40	146.93 ± 13.97	0.636
Albumin (g/dL; mean ± SD)	43.28 ± 3.34	43.22 ± 3.41	43.47 ± 3.11	0.498
Bilirubin (mg/dL; mean ± SD)	13.32 ± 4.94	13.31 ± 4.95	13.36 ± 4.96	0.916
Platelet count (× 10^3^/mm^3^; mean ± SD)	185.62 ± 72.00	184.02 ± 67.22	190.45 ± 84.93	0.422
Prothrombin time (INR; mean ± SD)	1.01 ± 0.08	1.02 ± 0.08	1.01 ± 0.08	0.264
HBV-DNA copy number (Log; mean ± SD)	2.46 ± 2.41	2.54 ± 2.42	2.22 ± 2.38	0.233
Bleeding [mL; median (IQR)]	200 (100–400)	200.00 (100–400)	275.00 (100–500)	0.834
Hospital stay (days; mean ± SD)	11.64 ± 3.87	11.67 ± 3.83	11.55 ± 3.98	0.774
Operation time (min; mean ± SD)	157.32 ± 58.95	155.02 ± 57.54	164.23 ± 62.77	0.160
Portal vein embolization [cases (%)]				1.000
Yes	143 (33.10)	107 (33.02)	36 (33.33)	
No	289 (66.90)	217 (66.98)	72 (66.67)	
Tumor size [cm; median (IQR)]	5.00 (3.00–8.00)	5.00 (2.95–8.00)	4.00 (3.00–8.00)	0.932
Tumor location [cases (%)]^a^				0.066
Central	270 (62.5)	194 (59.9)	76 (70.4)	
Non-central	162 (37.5)	130 (40.1)	32 (29.6)	
Tumor lesions [cases (%)]				0.375
1	320 (74.07)	236 (72.84)	84 (77.78)	
2–3	112 (25.93)	88 (27.16)	24 (22.22)	
Liver macronodular cirrhosis [cases (%)]				0.248
None	111 (25.69)	87 (26.85)	24 (22.22)	
Light	304 (70.37)	226 (69.75)	78 (72.22)	
Medium	14 (3.24)	8 (2.47)	6 (5.56)	
Heavy	3 (0.69)	3 (0.93)	0 (0.00)	
Cancer cell differentiation [cases (%)]				0.811
Low	213 (49.31)	162 (50.00)	51 (47.22)	
Medium	205 (47.45)	151 (46.60)	54 (50.00)	
High	14 (3.24)	11 (3.40)	3 (2.78)	
Tumor thrombus [cases (%)]				0.546
Present	36 (8.33)	29 (8.95)	7 (6.48)	
Absent	396 (91.67)	295 (91.05)	101 (93.52)	
MVI [cases (%)]				0.336
Present	175 (40.51)	136 (41.98)	39 (36.11)	
Absent	257 (59.49)	188 (58.02)	69 (63.89)	
Surgical procedure [cases (%)]				0.608
Irregular	263 (60.88)	200 (61.73)	63 (58.33)	
Regular	169 (39.12)	124 (38.27)	45 (41.67)	
Tumor encapsulation [cases (%)]				0.586
No	153 (35.42)	119 (36.73)	34 (31.48)	
Incomplete	109 (25.23)	79 (24.38)	30 (27.78)	
Complete	170 (39.35)	126 (38.89)	44 (40.74)	
Tumor boundary [cases (%)]				0.546
Clear	396 (91.67)	295 (91.05)	101 (93.52)	
Unclear	36 (8.33)	29 (8.95)	7 (6.48)	
8th AJCC T stage [cases (%)]				0.320
T1a	66 (15.28)	44 (13.58)	22 (20.37)	
T1b	142 (32.87)	104 (32.10)	38 (35.19)	
T2	157 (36.34)	123 (37.96)	34 (31.48)	
T3	65 (15.05)	51 (15.74)	14 (12.96)	
T4	2 (0.46)	2 (0.62)	0 (0.00)	
BCLC stage [cases (%)]				0.731
0	31 (7.18)	23 (7.10)	8 (7.41)	
A1	289 (66.90)	213 (65.74)	76 (70.37)	
A2	18 (4.17)	15 (4.63)	3 (2.78)	
B	94 (21.76)	73 (22.53)	21 (19.44)	
Follow-up time [months; median (IQR)]	14.38 (4.60–29.38)	13.58 (4.49–26.41)	23.10 (6.63–32.79)	0.012

*SD* standard deviation, *IQR* interquartile range, *AFP* alpha fetoprotein, *CA19-9* carbohydrate antigen 19-9, *AST* aspartate transaminase, *ALT* aminotransferase, *INR* international normalized ratio, *HBV* hepatitis B virus, *MVI* microvascular tumor invasion, *AJCC* American Joint Committee on Cancer, *BCLC* Barcelona Clinic Liver Cancer

^†^The difference between the training cohort and the validation cohort was compared using the Independent Samples *t* test or Mann–Whitney *U* test

^a^Central = section I, IV, V, VIII; Non-central = section II, III, VI, VII

### Development and validation of the nomogram model

**Fig. 1:**
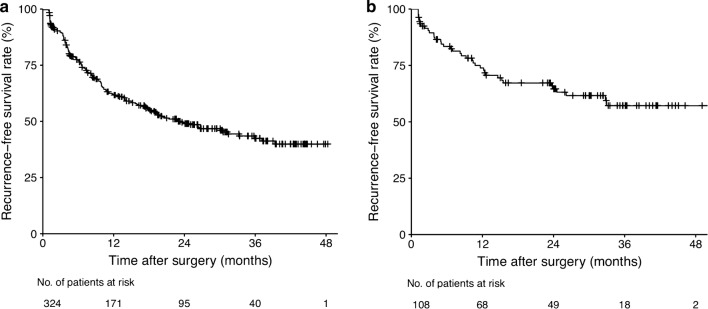
Kaplan–Meier estimates of recurrence-free survival in the training cohort (**a**) and the validation cohort (**b**) of patients who underwent laparoscopic hepatectomy for hepatocellular carcinoma

Kaplan–Meier estimates of RFS in the training and validation cohorts are presented in Fig. 1. In the training cohort, 156 (48.15%) patients developed recurrence during a median follow-up of 13.58 months (interquartile range [IQR], 4.49–26.41 months), and the 1-, 2-, and 3-year RFS rates were 62.1%, 49.0%, and 42.5% with a median RFS after primary LH of 23.6 months (Fig. 1a).

Univariate analyses (Table 2) revealed that positive HBsAg (*P *=0.008), presence of liver macronodular cirrhosis (*P *=0.006), elevated AFP (*P *<0.001), increased AST (*P *<0.001) and ALT (*P *=0.018), greater amount of bleeding (*P *=0.002), larger tumor size (*P *<0.001), 2–3 lesions (*P *<0.001), low cancer cell differentiation (*P *=0.001), presence of tumor thrombus (*P *<0.001), MVI (*P *<0.001), absence of tumor encapsulation (*P *=0.010), and unclear tumor boundary (*P *<0.001) were identified as significant prognostic factors for RFS. In multivariate analysis, HBsAg (hazard ratio [HR], 1.838; 95% confidence interval [CI] 1.016–3.327; *P *=0.044), tumor number (HR, 1.774; 95% CI 1.223–2.573; *P *=0.003), tumor thrombus (HR, 2.356; 95% CI 1.344–4.130; *P *=0.003), cancer cell differentiation (HR, 0.745; 95% CI 0.535–1.036; *P *=0.080), and the presence of MVI (HR, 1.673; 95% CI 1.150–2.433; *P *=0.007) were identified as independent predictors for RFS (Table 2).

**Table 2:** Cox proportional hazards regression analyses of recurrence in the training cohort

Variable	Total (cases)	Events (cases)	Univariate analysis		Multivariate analysis		
			HR (95% CI)	*P* value	Estimated coefficient	HR (95% CI)	*P* value
Age (years)			0.738 (0.471–1.156)	0.233			
< 65	281	139					
≥ 65	43	17					
Sex			0.966 (0.580–1.609)	0.895			
Male	285	140					
Female	39	16					
Hepatitis B surface antigen			2.070 (1.363–3.146)	0.008	0.609	1.838 (1.016–3.327)	0.044
Yes	276	142					
No	48	14					
Liver macronodular cirrhosis			1.741 (1.235–2.455)	0.006	0.125	1.133 (0.814–1.575)	0.460
None	87	33					
Light	226	121					
Medium	8	1					
Heavy	3	1					
AFP (ng/mL)			1.712 (1.242–2.361)	< 0.001	0.128	1.137 (0.793–1.629)	0.485
< 200	182	74					
≥ 200	142	82					
CA19-9 (ng/mL)			1.359 (0.920–2.009)	0.092			
< 35	248	116					
≥ 35	76	40					
AST (IU/L)			1.930 (1.226–3.039)	< 0.001	0.122	1.129 (0.673–1.895)	0.646
< 50	266	119					
≥ 50	58	37					
ALT (IU/L)			1.516 (1.039–2.213)	0.018	0.285	1.329 (0.866–2.041)	0.193
< 50	246	109					
≥ 50	78	47					
Hemoglobin (g/L)			0.733 (0.472–1.140)	0.124			
< 130	55	30					
≥ 130	269	126					
Albumin (g/dL)			0.819 (0.176–3.812)	0.778			
< 35	5	2					
≥ 35	319	154					
Bilirubin (mg/dL)			1.157 (0.770–1.737)	0.461			
< 17.1	260	124					
≥ 17.1	64	32					
Platelet count (*10^3^/mm^3^)			0.610 (0.250–1.490)	0.168			
< 80	14	8					
≥ 80	310	148					
Prothrombin time (INR)			–	0.070			
≤ 0.85	3	0					
0.85–1.2	313	151					
> 1.2	8	5					
HBV-DNA copy number (Log)			1.183 (0.863–1.621)	0.301			
< 2	142	64					
≥ 2	182	92					
Bleeding (mL)			2.139 (1.090–4.197)	0.002	0.550	1.734 (0.872–3.446)	0.117
< 800	299	138					
≥ 800	25	18					
Operation time (min)			1.194 (0.769–1.855)	0.400			
< 200	273	129					
≥ 200	51	27					
Portal vein embolization			1.407 (1.000–1.980)	0.400			
Yes	107	59					
No	217	97					
Tumor size (cm)			2.175 (1.570–3.012)	< 0.001	0.208	1.231 (0.809–1.872)	0.332
< 5	184	69					
≥ 5	140	87					
Tumor number			2.324 (1.592–3.392)	< 0.001	0.573	1.774 (1.223–2.573)	0.003
Single	236	94					
2–3 lesions	88	62					
Cancer cell differentiation			0.615 (0.446–0.849)	0.001	− 0.295	0.745 (0.535–1.036)	0.080
Low	162	90					
Medium	151	64					
High	11	2					
Tumor thrombus			4.126 (1.856–9.170)	< 0.001	0.857	2.356 (1.344–4.130)	0.003
Yes	29	23					
No	295	133					
MVI			2.291 (1.646–3.189)	< 0.001	0.514	1.673 (1.150–2.433)	0.007
Yes	136	85					
No	188	71					
Surgical procedure			1.108 (0.444–2.764)	0.718			
Irregular	200	97					
Regular	124	59					
Tumor encapsulation			0.978 (0.639–1.498)	0.010	− 0.079	0.924 (0.753–1.135)	0.453
Absent	119	63					
Incomplete	79	43					
Complete	126	50					
Tumor boundary			2.343 (1.204–4.559)	< 0.001	− 0.016	0.985 (0.535–1.814)	0.960
Clear	295	136					
Unclear	29	20					

*HR* hazard ratio, *CI* confidence interval, *AFP* = alpha fetoprotein, *CA19-9* carbohydrate antigen 19-9, *AST* aspartate transaminase, *ALT* aminotransferase, *INR* international normalized ratio, *HBV* hepatitis B virus, *MVI* microvascular tumor invasion

**Fig. 2:**
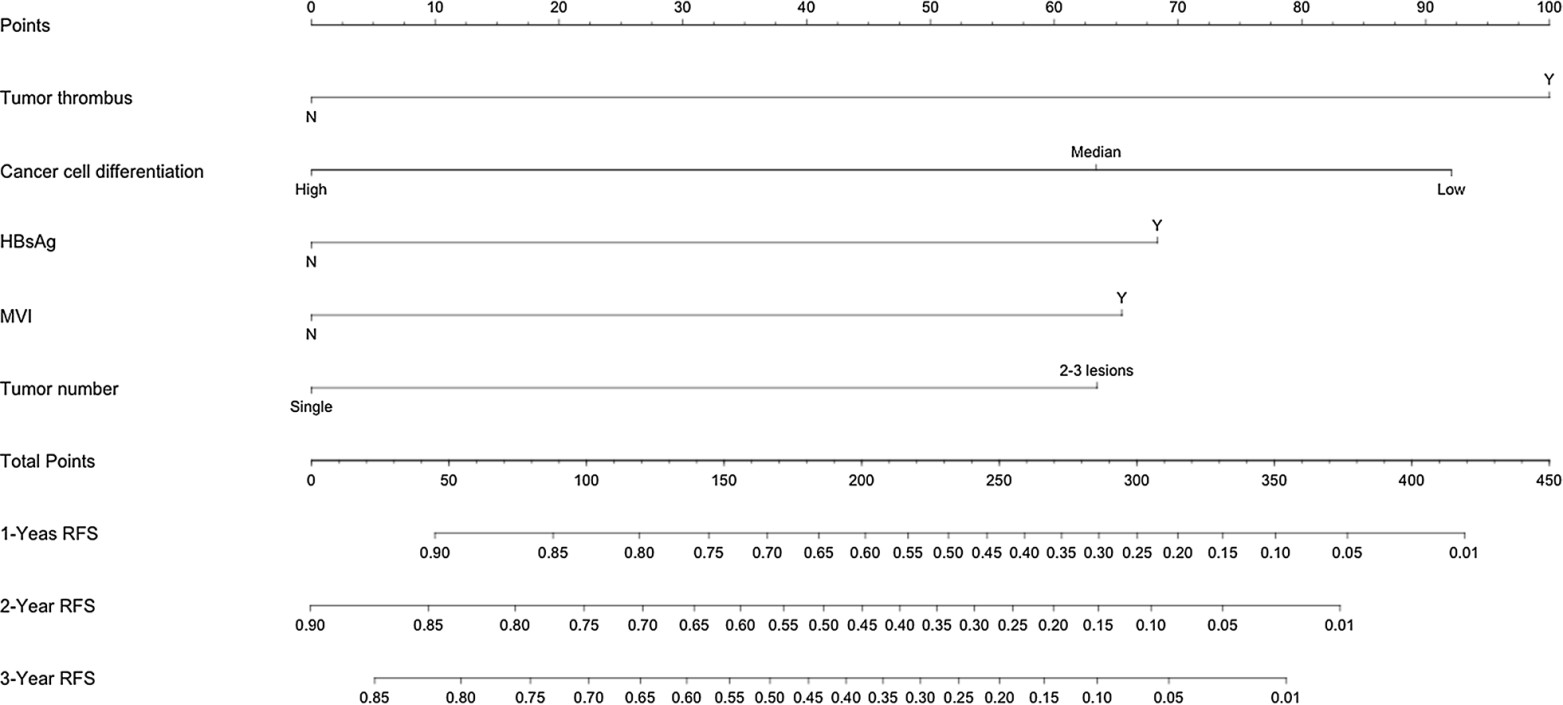
Nomogram depicting 1-, 2- and 3-year recurrence-free survival probability. By drawing a line between each variable and the uppermost component points, the appropriate points can be assigned to five variables. The sum of these five points can be expressed on the total point line. The 1-, 2-, and 3-year recurrence-free survival probability can be calculated by connecting each point to the survival line. The exact values of individual factors are tumor thrombus (100, 0 points), cancer cell differentiation (92, 63, 0 points), HBsAg (68, 0 points), MVI (65, 0 points), and tumor number (63, 0 points). *HBsAg* hepatitis B surface antigen, *MVI* microvascular tumor invasion, *RFS* recurrence-free survival

**Fig. 3:**
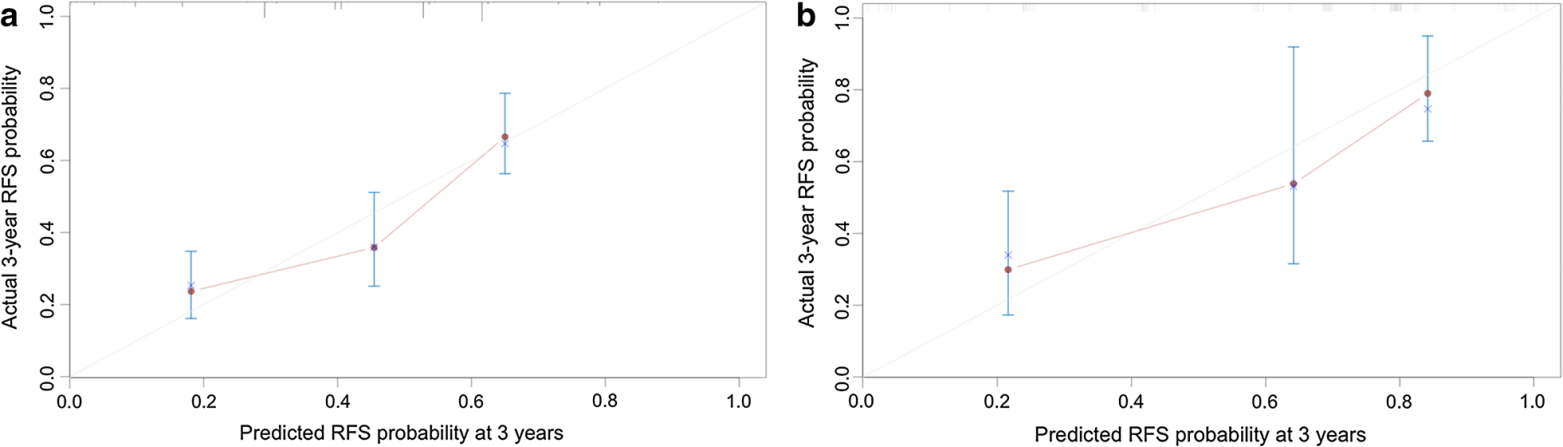
Calibration plots of recurrence-free survival in the training and validation cohorts. The calibration curves derived from the training (**a**) and validation (**b**) cohorts are almost a diagonal line that would represent perfectly reliable prediction

The prognostic nomogram that integrated all the independent prognostic factors for RFS derived from the training cohort is shown in Fig. 2. The C-index for RFS prediction in the training and validation cohorts were 0.703 (95% CI 0.747–0.659) and 0.789 (95% CI 0.858–0.719), respectively. The calibration plot for the RFS probability in the training and validation cohorts at 3 years after LH showed acceptable consistency between the prediction by nomogram and actual observation (Fig. 3).

### ROC analysis among nomogram, 8th AJCC TNM, and BCLC

**Fig. 4:**
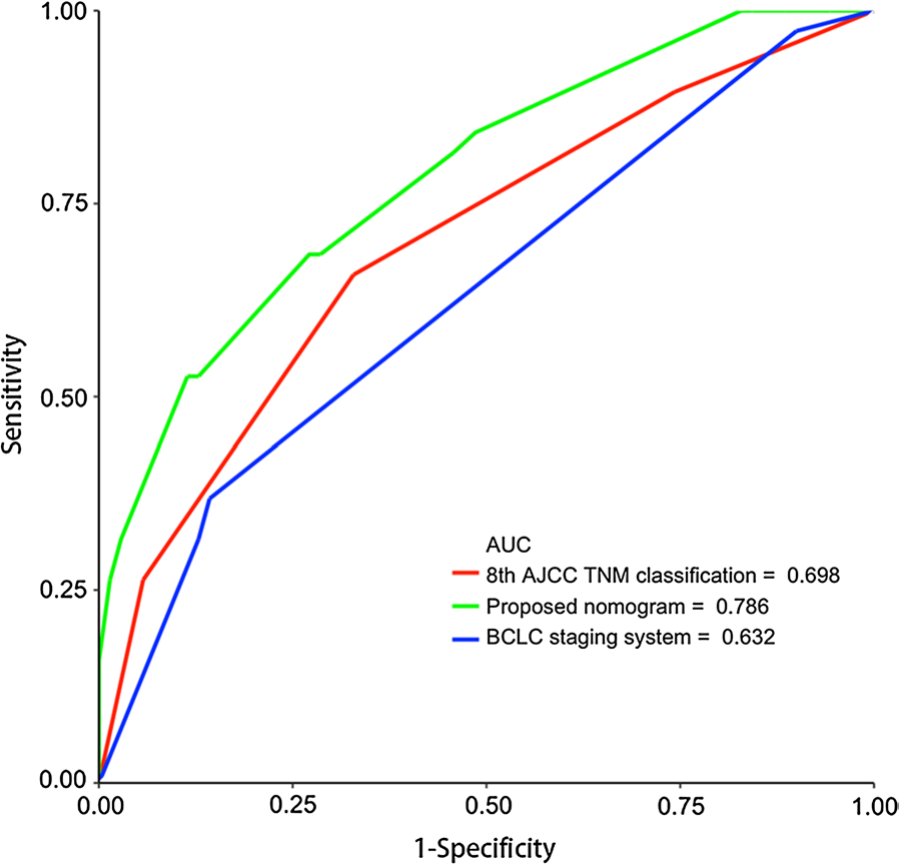
ROC analysis of recurrence-free survival at 3 years in the validation cohort using the proposed nomogram, the 8th AJCC TNM classification, and the BCLC staging system. The C-index value of the proposed nomogram was superior to the C-index values of the other two systems

We compared the accuracy and probability of our nomograms with the clinically used prognostic models, namely the 8th AJCC TNM classification and the BCLC staging system. ROC curves for the 3-year RFS were plotted for the 108 patients in the validation cohort. The discriminatory ability of the present nomogram model, which had a C-index corresponding to the area under the ROC curve of 0.786 (95% CI 0.698–0.875), was superior to that of the 8th AJCC TNM classification and the BCLC staging system with C-indexes of 0.698 (95% CI 0.596–0.799) and 0.632 (95% CI 0.542–0.722), respectively (Fig. 4). According to the ROC analysis, we observed an improved predictive benefit in RFS and higher threshold probability when using our proposed nomogram as compared to the other predictive systems.

## Discussion

In the present study, we developed and validated a practical nomogram model, based on clinicopathological characteristics of HCC patients who underwent LH, to predict the 1-, 2-, and 3-year RFS. It demonstrated superior prognostication performance compared with the 8th AJCC TNM classification and the BCLC staging system (C-index, 0.786 vs. 0.698 vs. 0.632, respectively).

It is widely believed that poor liver function and heavy tumor burden are significant prognostic factors that are associated with tumor recurrence after hepatectomy in HCC patients [15–17]. Compared to non-resection treatment, such as radiofrequency ablation, interventional therapy, and radiotherapy, establishing staging systems that are based on postoperative pathology combined with clinical factors seems more reliable for the prediction of recurrence, as it possesses more accurate and reliable information on tumor profiles as to that provided solely by postoperative pathology. However, almost all previously established staging systems are based on conventional hepatectomy which is much invasive to patients, and predictive model based per-patient is limited [18, 19]. Traditional opinions suggest that the evaluation of conventional hepatectomy is more depended on liver profiles that contribute more for long-term survival [18], whereas laparoscopic hepatectomy has comparable clinical outcomes to conventional hepatectomy and is less invasive, thereby reducing the injury to liver function for patients who are subjected to hepatectomy [20]. Simultaneously, this change of surgical selection increases the role of tumor burden in the prediction of recurrence for patients who are treated with LH. As a result, LH has different intrinsic properties from that of open procedure, and their long-term outcomes need to be separately mapped.

Several studies have emphasized the critical roles of tumor burden, gender, liver function, and performance status in the prognosis of HCC, but few have actually shown the role of detailed information of pathology in prognosis prediction [12, 21]. It is commonly supposed that the 8th AJCC TNM classification is one of the most prevalent staging systems of HCC, which is composed of TNM stage. However, this only classifies tumor burden and is limited in the power of prediction for HCC patients who are subjected to LH [22]. HCC patients who undergo surgical resection rarely suffer from lymph node metastases or distant metastases, and this classification thereby influences the accurate evaluation of RFS. The BCLC staging system takes both the liver function and tumor characteristics into account, including tumor extension, reserved liver function, physical status, and cancer-related symptoms [23]. The notable feature that distinguishes the BCLC staging system from other systems is the treatment recommendations for each stage based on the best treatment options currently available [24]. However, the BCLC class B (intermediate stage) covers a considerable heterogeneous population of HCC patients with varying degree of tumor extension, reserved liver function, and disease etiology, thus resulting in prognostic heterogeneity and preventing the decision of optimal treatment regimen selection. Meanwhile, the guidelines mentioned above are mainly based on preoperative clinical data or pathological information. Moreover, no guideline tailors for resectable HCC patients who were subjected to LH. Therefore, it is urgent to introduce a reliable, practicable, and individualized predictive model for patients who are candidates for surgical hepatectomy, especially LH.

The present nomogram integrates five independent risk factors for RFS, including HBsAg, tumor thrombus, tumor number, cancer cell differentiation, and MVI. Many studies have indicated that HBV infection, tumor thrombus, and MVI were significant risk factors for recurrence in patients with HCC [25–27]. The underlying hepatitis background was significantly associated with late recurrence and multicentric carcinogenesis. Tang et al. [27] reported that HBV infection might accelerate hepatocarcinogenesis via the integration of HBV DNA into the host genome, and continuous expression of viral proteins such as HBx might be involved in hepatocarcinogenesis. However, the major source of early recurrence is generally thought as metastasis, which is mainly derived from vascular invasion. Hirokawa et al. [25] indicated that circulating tumor cells were closely related to epithelial–mesenchymal transition and mesenchymal–epithelial transition which are the significant property of cancer stem cells. Given the early diagnosis of HCC, tumor thrombus is rare. Alternately, MVI is another potent parameter indicating vascular invasion for the prediction of recurrence [28]. Additionally, the present study demonstrated that tumor number predicted HCC recurrence, which was consistent with the results of other studies [29, 30]. However, tumor size could not be included in the nomogram proposed for HCC recurrence prediction in the present study, although other studies have indicated its insightful role in prognostic prediction [31, 32]. The possible explanation is that vascular invasion plays a more critical role in recurrence than tumor size, especially for patients with tumor size > 2 cm, according to 8th AJCC TNM classification [14, 33]. Besides, surgical margin was also not included in the nomogram, and it could be explained in the way that the patients included in the present study all had a minimal surgical margin of 1 cm, which indicated better RFS [13]. Interestingly, cancer cell differentiation was found to be a significant prognostic factor, and this was rarely mentioned in other studies. Low cancer cell differentiation has been reported to be the property of cancer progenitor or cancer stem cells which has high malignant biological behavior [34].

As the clinical and pathological factors mentioned in the present study have been validated separately in previous conventional hepatectomy studies, and the present study is the first to combine them together to assess patients who are subjected to laparoscopic hepatectomy. Hence, the proposed nomogram can be used to better guide routine follow-up for patients who have undergone LH as initial therapy. Patients characterized with a high recurrence score on our nomogram could be counseled to receive more high-end imaging examinations and close follow-up. In addition, more aggressive adjuvant therapy might be proposed, even if the results of the latest postoperative examinations indicated no evidence of recurrence. Conversely, the follow-up period for low-risk patients should refer to the clinical guideline [3].

Although our nomogram demonstrated satisfactory performance compared with existing systems used clinically, its related limitations need to be described. First, the nomogram was derived from data collected at a single institution, and the follow-up duration was relatively short for prognosticating long-term survival outcomes. Second, as this is a retrospective study for predicting the anticipated result, our nomogram needs to be confirmed in a prospective cohort. Third, our nomogram is mainly based on pathological outcomes, therefore, it is inapplicable to evaluate non-surgical patients.

## Conclusions

We proposed a nomogram for predicting the postoperative RFS for HCC patients who underwent LH, based on easy-to-obtain clinical factors, comprising of HBsAg, tumor thrombus, tumor number, cancer cell differentiation, and MVI. The nomogram demonstrated relatively higher prediction than conventional 8th AJCC TNM and BCLC staging systems, and these findings, after proper validation, can be used as a model to better to guide individualized post-LH surveillance protocols of such patients.

## Data Availability

The datasets analyzed during the current study are available in the Research Data Deposit (RDD) repository (http://www.researchdata.org.cn/; RDD Number: RDDA2019001113).

## Abbreviations

HCC: hepatocellular carcinoma
LH: laparoscopic hepatectomy
RFS: recurrence-free survival
AJCC: American Joint Committee on Cancer
BCLC: Barcelona Clinic Liver Cancer
SYSUCC: Sun Yat-sen University Cancer Center
HBsAg: hepatitis B surface antigen
ALT: alanine aminotransferase
AST: aspartate transaminase
ALB: albumin
AFP: alpha fetoprotein
CA 19-9: carbohydrate antigen 19-9
HGB: hemoglobin
MVI: microvascular tumor invasion
CT: computed tomography
MRI: magnetic resonance imaging
ROC: receiver operating characteristic
IQR: interquartile range
HR: hazard ratio
IC: confidence interval
